# Randomized clinical trial evaluating kinetic benefits of desensitizing agents: Magnitude, onset, and stability of relief

**DOI:** 10.1002/JPER.24-0688

**Published:** 2025-05-30

**Authors:** Aaron R. Biesbrock, Tao He, Yuanshu Zou, Julie M. Grender, Pejmon Amini, Paul A. Sagel, Andrew Groth, Malgorzata Klukowska

**Affiliations:** ^1^ Global Oral Care Research & Development The Procter & Gamble Company Mason Ohio USA; ^2^ Silverstone Research Group Las Vegas Nevada USA

**Keywords:** dentin desensitizing agents, dentinal fluid, oral hygiene, oxalates, potassium nitrate, stannous fluoride

## Abstract

**Background:**

Dentin hypersensitivity (DH) is common, particularly among periodontal patients. This trial evaluated magnitude of desensitizing benefit, onset of action, and complete relief of three dentifrices with distinct desensitizing technologies.

**Methods:**

Healthy adults with DH were enrolled and randomized to one of four dentifrices in this randomized, controlled, double‐blind trial: marketed potassium nitrate (KNO_3_), marketed stannous fluoride (SnF_2_), experimental 1.5% oxalate, or negative control (NC) monofluorophosphate. Participants brushed with their assigned dentifrice twice daily for 8 weeks, followed by 3 weeks during which all participants used the NC. DH was measured by thermal challenge (Schiff Index) and tactile challenge (Yeaple Probe) at baseline, Day 3, and Weeks 2, 4, 8, and 11.

**Results:**

A total of 120 participants were randomized to treatment, and 118 completed the study. *Thermal challenge (Schiff)*: All treatments provided greater relief than NC at all timepoints (*p* ≤ 0.004). The SnF_2_ dentifrice had greater thermal improvements than KNO_3_ dentifrice at Week 2 (*p* = 0.021) and in a random coefficients model across all timepoints (*p* = 0.015). The percentage of participants showing complete relief in at least one test tooth was greater for SnF_2_ versus KNO_3_ through Week 8 (all *p* ≤ 0.044). *Tactile challenge* (*Yeaple Probe)*: All treatments provided greater tactile sensitivity relief than NC at all timepoints (*p* ≤ 0.019), except KNO_3_ at Day 3 (*p* = 0.197). The SnF_2_ dentifrice had greater improvements in tactile scores versus the KNO_3_ dentifrice at Day 3 (*p* = 0.020).

**Conclusion:**

SnF_2_, KNO_3_, and oxalate dentifrices significantly reduced DH over 8 weeks. SnF_2_ dentifrice showed the greatest benefits for onset of relief, magnitude of benefit, and complete relief.

**Trial Registration:**

NCT03965039

**Plain language summary:**

This study tested four toothpastes for sensitivity relief, including speed and completeness of relief, among approximately 120 adults with sensitive teeth. They were randomly assigned to use one of four toothpastes: commercial toothpaste with potassium nitrate, commercial toothpaste with stannous fluoride, experimental toothpaste with oxalate, or standard fluoride toothpaste (control) with no antisensitivity ingredient. Participants used their toothpaste twice daily for 8 weeks, and then everyone used the control toothpaste for 3 weeks. Sensitivity was measured at the beginning of the study, after 3 days, and after 2, 4, 8, and 11 weeks by blowing cold air on sensitive teeth and moving a probe across the sensitive area (tactile assessment). The potassium nitrate, stannous fluoride, and oxalate toothpastes reduced sensitivity better than the control toothpaste at all times, except potassium nitrate toothpaste at Day 3 for tactile sensitivity. Stannous fluoride toothpaste reduced cold air sensitivity better than potassium nitrate toothpaste at Week 2 and across all times combined. More participants showed complete relief from cold air sensitivity for stannous fluoride toothpaste than potassium nitrate toothpaste through Week 8. Stannous fluoride toothpaste had greater improvements in tactile sensitivity versus potassium nitrate toothpaste at Day 3. Overall, stannous fluoride toothpaste was superior at relieving sensitivity.

## 1 INTRODUCTION

Dentin hypersensitivity is a common condition, with prevalence measures varying widely (1.3%–92.1%).^1^ Notably, dentinal hypersensitivity is reported to be high among periodontal patients (60%–90%).^2^, ^3^ The variability likely results from differences in populations and methods used in investigations, which include direct clinical examinations or professional/patient‐focused questionnaires.^4^, ^5^, ^6^, ^7^ When presenting in dental offices, patients frequently complain of dental pain. The pain associated with dentin hypersensitivity is characterized by short, sharp pain arising from exposed dentine in response to stimuli.^8^ Dental professionals often attribute the source of pain to dentin hypersensitivity once other sources (e.g., dental caries, cracked‐tooth syndrome, defective restorations) are excluded.

**FIGURE 1:**
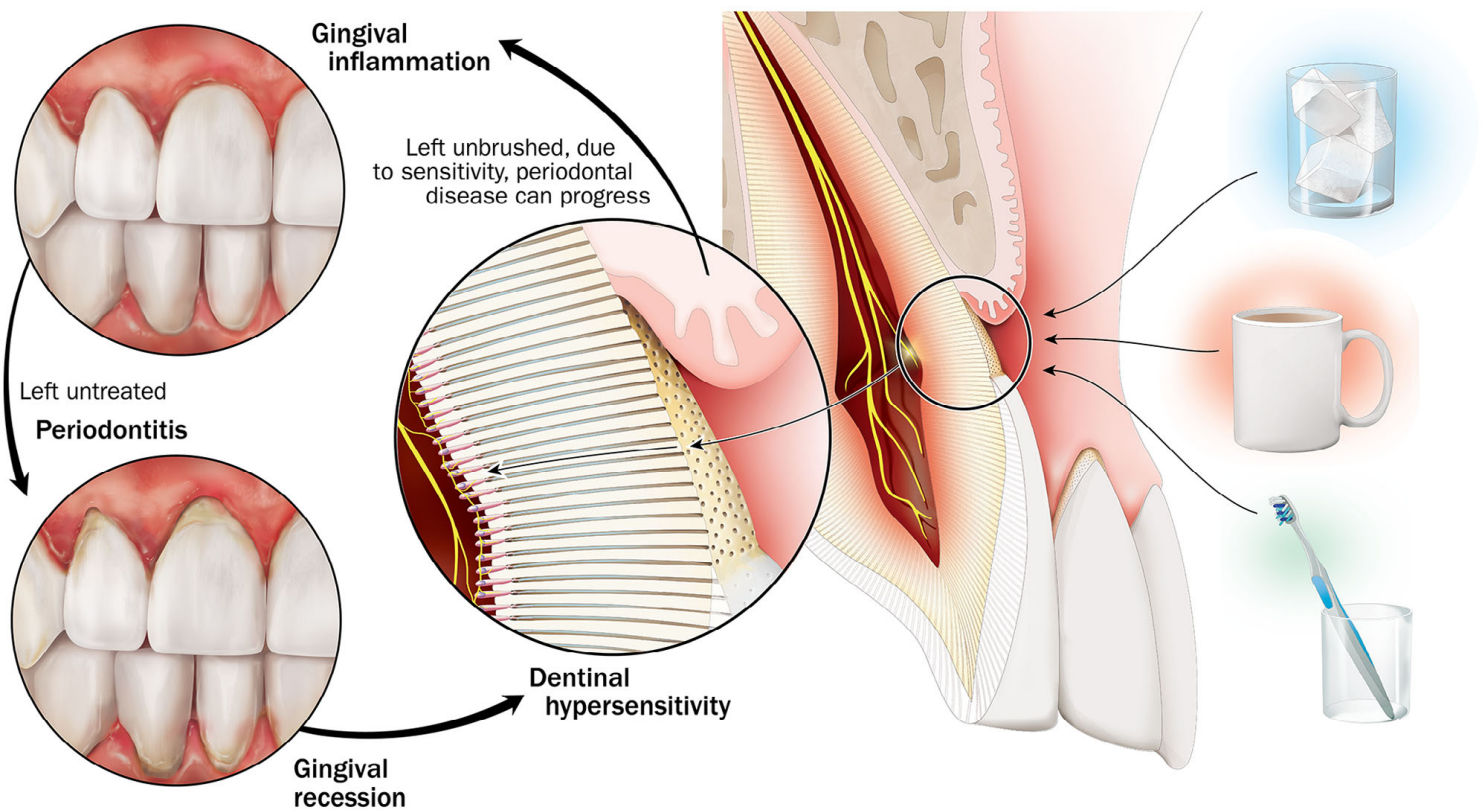
Effects of dentinal hypersensitivity.

Many theories have been suggested to explain the mechanism involved in the etiology of dentin hypersensitivity.^9^, ^10^ The *hydrodynamic theory* by Brännström and colleagues is now generally accepted as the mechanism for this condition.^11^ The hydrodynamic theory comprises three essential components: exposure of dentin to the oral cavity, the development of “patent” (open) tubuli on the dentin surface, and excitation of pulp nerves through mechanical receptors to oral stimuli. Risk factors for dentin exposure include gingival recession, typically due to periodontal disease or aggressive toothbrushing, and erosive tooth wear^12^ (see Figure 1). Once dentin and dentinal tubuli are exposed to the oral environment, an oral stimulus can cause dentinal tubuli to be filled with fluid arising from the pulp. The subsequent fluid movement in the exposed dentinal tubuli stimulates neuromechanical receptors in the tooth pulp, which are sensitive to fluid pressure, resulting in activation of the pulpal nerves causing the pain response. Stimuli associated with pain responses include thermal, chemical, tactile, or hygroscopic (thick sweet and sour fluids), all of which can provoke fluid movement within dentin.

The mechanistic understanding and acceptance of the hydrodynamic theory has prompted considerable translational research into potential therapies, including professionally applied therapies as well as over‐the‐counter products. Treatments are generally focused on two aspects of the Brännström model—suppression of nerve response to stimuli or sealing tubuli at the dentin surface. Professional treatments center on providing immediate relief by sealing dentin tubuli with restorative materials (e.g., fluoride varnishes, resin, glass ionomer cements, composites) or application of lasers.^13^, ^14^, ^15^, ^16^ These treatments are generally effective, but may be expensive and resource‐consuming for professional staff.

As an alternative or adjunct to professional treatment, dental professionals often recommend over‐the‐counter desensitizing products.^17^ Similar to professional treatment, these products work by tubule occlusion or suppression of nerve response to stimuli. One approach involves creating artificial smear layers on dentin surfaces through physical or chemical action. To varying degrees, a number of technologies have shown clinical efficacy with this approach including those based on strontium salts, zinc salts, oxalate salts, bioactive calcium phosphate glasses, stannous fluoride (SnF_2_), and SnF_2_ plus arginine.^17^, ^18^, ^19^ Another set of treatments with reported successful clinical effects include toothpastes containing potassium nitrate (KNO_3_).^17^, ^18^, ^19^, ^20^ Researchers hypothesize that KNO_3_ depolarizes the nerves in pulp, suppressing pulp responses to stimuli.^20^

Research^21^, ^22^ suggests that important needs of patients with dentin hypersensitivity include speed of relief (onset), protection against differing common stimuli (viz. hot/cold or tactile), and the magnitude of benefit. The current dentin hypersensitivity study was designed to compare SnF_2_, KNO_3_, and oxalate technologies with respect to these efficacy parameters, as well as durability of benefit.

## 2 MATERIALS AND METHODS

This was a randomized, controlled, single‐center, double‐blind clinical trial to assess efficacy and safety of three dentifrices with different desensitizing technologies and a negative control dentifrice over 8 weeks of use, followed by 3 weeks of washout product (negative control) use to assess durability of relief. The study was conducted by the Silverstone Research Group, an American Dental Association (ADA)‐qualified independent research site in Las Vegas, Nevada, from July 2019 to October 2019 in accordance with the Helsinki Declaration as revised in 2013. The ADA qualification process involves inspection of the clinical facility by an independent team of dental experts who review the accuracy of data collection procedures and the experience and training of directors and staff to ensure the research site is fully independent, follows necessary protocols, and produces accurate, valid, and reliable data. All participants were provided written informed consent. The IntegReview Institutional Review Board approved the study protocol (2019079). The protocol followed core principles of the ADA Seal of Acceptance guidelines for dentin hypersensitivity products, with additional rigor including longer duration, more study visits, and a post‐treatment durability evaluation.^23^

### 2.1 Eligibility criteria

Generally healthy adults with a history of tooth sensitivity were evaluated for enrollment. For study inclusion, participants had to provide written informed consent, agree to follow all study procedures and only use test products provided for oral hygiene, agree not to participate in other dental product studies or receive any dental treatment during the study, and have two teeth with a thermal (Schiff) sensitivity score >1 and a tactile (Yeaple Probe) score of 10–20 g. Exclusion criteria were evidence of chronic medical conditions or drug use associated with daily pain; dental prophylaxis within 2 weeks of baseline visit; professional or at‐home tooth bleaching within 4 weeks of baseline visit; use of professional or over‐the‐counter desensitizing treatment within 6 weeks of baseline visit; periodontal surgery, orthodontic treatment, or restorations in the preceding 3 months; teeth with pathology likely to cause pain; self‐reported pregnancy or lactation; or any condition predisposing participants to dentin hypersensitivity or that might interfere with safe study participation.

### 2.2 Study design

At the screening/baseline visit, demographic information and entrance criteria were assessed. A comprehensive oral examination was conducted of the oral and perioral region, including hard and soft tissues. Tactile sensitivity was assessed using a standard calibrated Yeaple Probe beginning at 10 g probing force.^7^ Approximately 5 min later, thermal sensitivity was assessed using a standard cold air thermal challenge (Schiff Index).^6^ For each participant, two test teeth were selected by the clinician having a thermal score >1 and tactile score between 10 and 20 g. If the qualifying eligible teeth were in the same quadrant, the two test teeth were required to be separated by at least two other teeth. Eligible participants were stratified based on their pretreatment thermal and tactile sensitivity scores, age, and sex. Within strata, participants were randomly assigned to a treatment group using an encoded program supplied by the sponsor. The assignment process and distribution of test products were conducted by site staff in a protected area to ensure blinding of the examiner to the test product identity.

The four treatment groups were experimental 1.5% oxalate dentifrice*, marketed 5% KNO_3_ dentifrice†, marketed 0.454% SnF_2_ dentifrice‡, or negative control anticavity dentifrice.§ Subtle differences existed between dentifrice in terms of appearance and taste. In order to maintain the integrity of the double blind, dentifrice was packaged in identical white uniform overtubes to conceal any potential differences. All participants received a kit box containing the disguised dentifrice, an Oral‐B‖ Indicator soft manual toothbrush, a timer, and identical instructions for use. All kit boxes were identically sized and had identical box labels, except for a unique kit number used for assignment. All participants were instructed to use only the assigned test products in place of normal at‐home oral hygiene twice daily for 1 min per brushing for the first 8 weeks of the study. Participants completed their first brushing supervised onsite, followed by a post‐treatment oral examination approximately 5 min post brushing. Adverse events, if any, were recorded at all visits.

Participants returned to the site after 3 days and at Weeks 2, 4, and 8 for efficacy and safety assessments, following the same procedures conducted at baseline. Following Week 8, kit boxes were returned and all participants were supplied with blinded negative control washout dentifrice to use for 3 weeks to evaluate durability of hypersensitivity relief. Participants returned to the site at Week 11 with their kit box. Site staff visually inspected product upon return as a compliance check. Efficacy and safety evaluations were conducted following procedures used at other visits.

### 2.3 Clinical measures

#### 2.3.1 Thermal challenge (Schiff Index)^6^

A one‐second application of cold air was delivered onto each tooth tested from a standard dental unit syringe. A score was recorded ranging from 0 to 3 according to the established Schiff Sensitivity Index Scale: 0 = participant does not respond to stimulus; 1 = participant responds to stimulus, but does not request discontinuation of stimulus; 2 = participant responds to stimulus and requests discontinuation or moves from stimulus; and 3 = participant responds to stimulus, considers stimulus to be painful, and requests discontinuation of the stimulus.

#### 2.3.2 Tactile challenge (Yeaple Probe)^7^

Sensitivity to tactile stimuli was assessed by application of a calibrated transducer modified periodontal probe (Yeaple Probe). A #16 explorer tip was used with the Yeaple Probe, which was kept perpendicular to the root surface of the test tooth. The tip was moved horizontally across the facial surface over the sensitive area. Two horizontal sweeps were made at each force setting. Testing began at 10 g and increased by 10 g to a maximum of 50 g. Each successive challenge increased until a “yes” pain response was repeated. If a second “yes” was not obtained, the force setting was increased and continued until a force elicited two consecutive “yes” pain responses, which was recorded as the threshold. If no sensitivity was found up to 50 g, 50 g was recorded as the threshold.

### 2.4 Safety

Safety was assessed by the absence of irreversible side effects. An oral soft tissue assessment was conducted via a visual examination of the oral cavity and perioral area utilizing a standard dental light, dental mirror, and gauze. The structures examined included the gingiva (free and attached), hard and soft palate, oropharynx/uvula, buccal mucosa, tongue, floor of the mouth, labial mucosa, mucobuccal/mucolabial folds, lips, and perioral area. An oral hard tissue assessment was conducted via a visual examination of the dentition and restorations utilizing a standard dental light, dental mirror, and air syringe. All abnormal findings were recorded and categorized by their location. An adverse event was recorded if a new abnormal finding was noted after product distribution or any previously noted abnormal finding increased in severity during the treatment period.

### 2.5 Statistical analysis

Power analyses were conducted with a two‐sided test (*α* = 0.05). Twenty‐eight participants per group completing this trial were expected to provide at least 90% power to detect a difference in mean thermal (Schiff) scores of at least 0.80 units between treatments assuming the variability(s) of thermal score is 0.88. Assuming the variability(s) of tactile (Yeaple Probe) score is 12.44, a sample size of 28 participants per group was expected to provide at least 90% power to detect a difference in tactile score of at least 10.97 units between treatments. Group size was determined utilizing data from a similarly designed study.

Thermal and tactile scores were averaged among test teeth within each participant for each assessment before and after treatment. For each treatment group and assessment, mean post‐treatment assessments were compared to pretreatment using a paired‐difference *t*‐test. For each post‐treatment assessment, mean comparisons between treatment groups were analyzed using analysis of covariance (ANCOVA) with the baseline score as a covariate. Statistical comparisons utilized two‐sided testing with a 5% significance level. Summary statistics (e.g., means, standard deviations, frequencies, etc.) of the demographic characteristics as well as efficacy measurements at baseline were calculated and compared between treatment groups using analysis of variance (ANOVA), chi‐square test, or Fisher's exact test. Multiplicity concerns were managed by prespecifying the thermal challenge measurement (Schiff Air Index) at Week 8 for actives versus negative control as the primary end point. All other comparisons were secondary analyses performed to characterize the kinetic onset on the sensitivity benefit.

To account for individual variability and capture potential heterogeneity in treatment effects, a random coefficients model with random intercept and random slope terms was utilized to assess the thermal/tactile score across time during the treatment period, with treatment, baseline score, week, and the interaction between treatment and week in the model.

## 3 RESULTS

One hundred and twenty‐eight participants were enrolled, 120 were randomized, and 118 completed the clinical study. Two participants voluntarily withdrew from the trial. Baseline characteristics were not statistically significantly different between treatment groups (*p* ≥ 0.05), as shown in Table 1.

**TABLE 1:** Baseline demographic and clinical characteristics.

Demographic/statistic or category	Negative control (*n* = 30)	Stannous fluoride (*n* = 30)	Oxalate (*n* = 30)	Potassium nitrate (*n* = 30)	Overall (*n* = 120)	*p* value
**Age (years)**						
Mean (SD)	39.03 (11.04)	38.73 (11.52)	39.87 (10.42)	42.53 (13.28)	40.04 (11.56)	0.575^a^
Min.–max.	20–60	22–65	22–65	19–69	19–69	
**Ethnicity**						
Hispanic or Latino^b^	6 (20%)	5 (17%)	9 (30%)	7 (23%)	27 (23%)	0.643^c^
Not Hispanic or Latino^b^	24 (80%)	25 (83%)	21 (70%)	23 (77%)	93 (78%)	
**Race**						
American Indian or Alaskan Native^b^	1 (3%)	0 (0%)	0 (0%)	0 (0%)	1 (1%)	0.050^d^
Asian^b^	2 (7%)	5 (17%)	1 (3%)	1 (3%)	9 (8%)	
Black or African American^b^	7 (23%)	3 (10%)	7 (23%)	2 (7%)	19 (16%)	
Multiracial^b^	5 (17%)	1 (3%)	7 (23%)	3 (10%)	16 (13%)	
Native Hawaiian or Other Pacific Islander^b^	0 (0%)	1 (3%)	0 (0%)	0 (0%)	1 (1%)	
White/Caucasian^b^	15 (50%)	20 (67%)	15 (50%)	24 (80%)	74 (62%)	
**Sex**						
Female^b^	21 (70%)	22 (73%)	22 (73%)	21 (70%)	86 (72%)	0.983^c^
Male^b^	9 (30%)	8 (27%)	8 (27%)	9 (30%)	34 (28%)	
**Sensitivity scores**						
Thermal challenge (Schiff) score	2.52 (0.382)	2.45 (0.379)	2.45 (0.356)	2.40 (0.381)	2.45 (0.372)	0.690^a^
Tactile challenge (Yeaple Probe) score	11.00 (2.754)	10.33 (1.826)	10.50 (2.013)	10.50 (1.526)	10.58 (2.069)	1.000^a^

^a^ Two‐sided analysis of variance *p* value for treatment comparison.

^b^ Number (percent) of participants in each category.

^c^ Two‐sided chi‐square *p* value for treatment comparison.

^d^ Two‐sided Fisher's exact test *p* value for treatment comparison.

### 3.1 Thermal challenge (Schiff Index) sensitivity results

At Day 3, the SnF_2_, oxalate, and KNO_3_ treatment groups demonstrated statistically significant reductions (*p* < 0.001) from baseline in thermal scores. At Weeks 2, 4, 8, and 11, all four groups demonstrated statistically significant reductions (*p* ≤ 0.007) from baseline in thermal scores.

**FIGURE 2:**
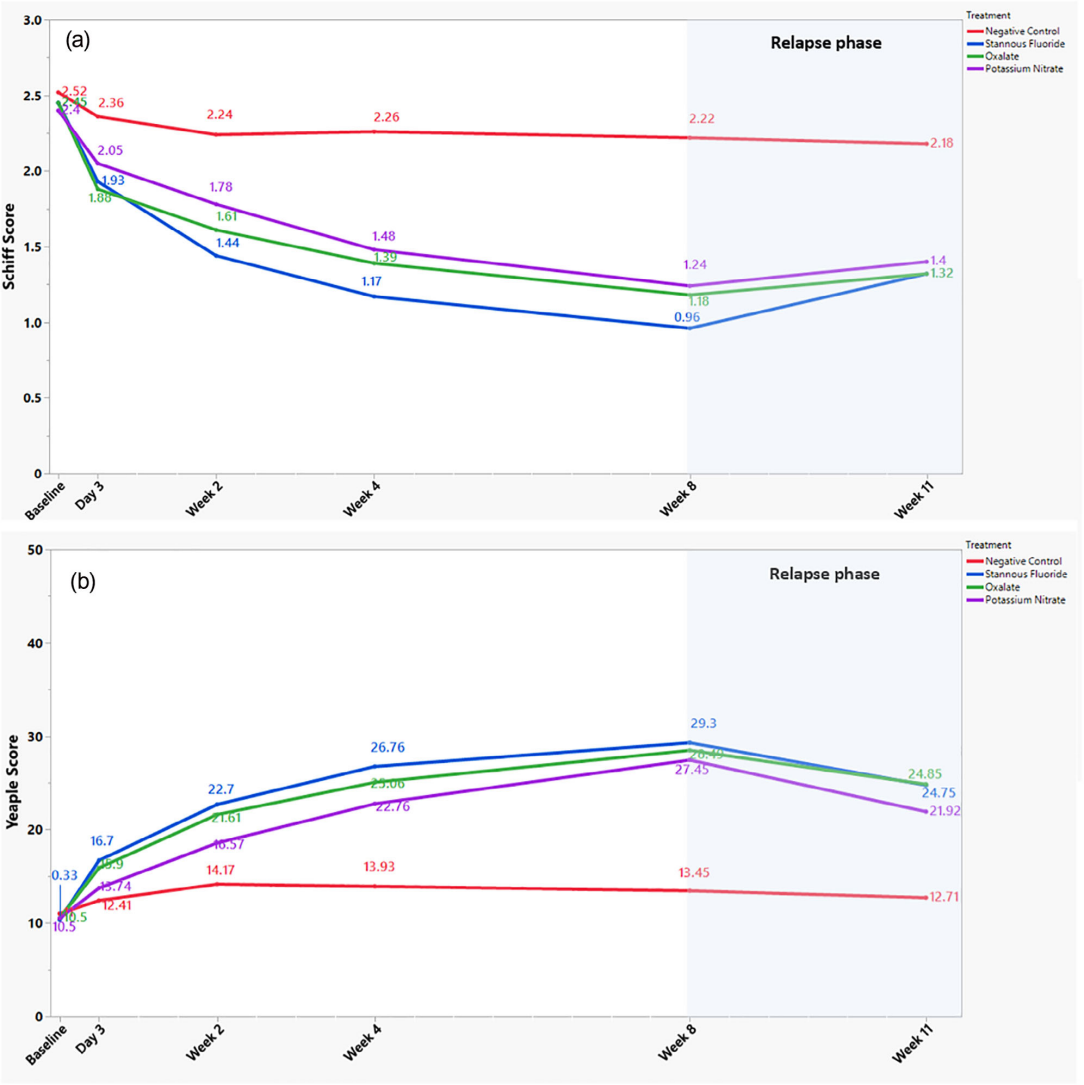
(a) Mean thermal challenge (Schiff Air Index) results. (b) Tactile challenge (Yeaple Probe) results.

Table 2A shows between‐treatment thermal results at all timepoints. All three treatments produced statistically significant improvements in thermal sensitivity at Day 3 versus the negative control (*p* ≤ 0.004), with tubule‐occluding treatments including SnF_2_ and experimental oxalate producing the largest effects (*p* < 0.001 for both). These trends continued through Week 2 where all three treatments showed significant reductions in thermal sensitivity versus negative control (*p* < 0.001). At Week 2, the SnF_2_ treatment also produced statistically significantly improvements compared to the KNO_3_ treatment (*p* = 0.021). These benefits continued for treatments through Weeks 4 and 8, with all three active treatments producing significant reductions in thermal sensitivity, measuring up to 57% for SnF_2_, 47% for oxalate, and 44% for KNO_3_ (*p* < 0.001 for all). At Week 11, when all participants had been using the negative control for 3 weeks, results showed statistically significant durability of benefits in thermal sensitivity for all three treatment groups (*p* < 0.001), with an improvement versus the negative control of 39.3% for SnF_2_, 39.6% for oxalate, and 35.8% for KNO_3_ (*p* < 0.001 for all). Thermal threshold measures are graphically portrayed in Figure 2a.

**TABLE 2A:** Thermal challenge (Schiff Air Index) results by visit. Lower scores indicate less sensitivity.

						*p* value^a^		
Treatment	*n*	Adjusted mean (SE)	% improvement from negative control	% improvement from stannous fluoride	% improvement from oxalate	Stannous fluoride	Oxalate	Potassium nitrate
**Day 3 (baseline mean = 2.450)**								
Negative control	29	2.36 (0.062)				<0.001	<0.001	0.004
Stannous fluoride	30	1.93 (0.081)	18.2%				0.673	0.317
Oxalate	30	1.88 (0.085)	20.3%	2.59%				0.165
Potassium nitrate	30	2.05 (0.085)	13.1%	−6.12%	−8.94%			
**Week 2 (baseline mean = 2.454)**								
Negative control	30	2.24 (0.074)				<0.001	<0.001	<0.001
Stannous fluoride	30	1.44 (0.117)	35.9%				0.274	0.021
Oxalate	29	1.61 (0.103)	28.2%	−12.0%				0.206
Potassium nitrate	30	1.78 (0.092)	20.4%	−24.2%	−10.9%			
**Week 4 (baseline mean = 2.449)**								
Negative control	30	2.26 (0.066)				<0.001	<0.001	<0.001
Stannous fluoride	30	1.17 (0.141)	48.3%				0.216	0.064
Oxalate	28	1.39 (0.116)	38.3%	−19.5%				0.565
Potassium nitrate	30	1.48 (0.087)	34.6%	−26.6%	−5.99%			
**Week 8 (baseline mean = 2.458)**								
Negative control	30	2.22 (0.059)				<0.001	<0.001	<0.001
Stannous fluoride	30	0.96 (0.144)	57.1%				0.258	0.104
Oxalate	29	1.18 (0.134)	47.0%	−23.4%				0.719
Potassium nitrate	29	1.24 (0.094)	44.3%	−29.6%	−5.01%			
**Week 11 (baseline mean = 2.458)**								
Negative control	30	2.18 (0.071)				<0.001	<0.001	<0.001
Stannous fluoride	30	1.32 (0.147)	39.3%				0.982	0.687
Oxalate	29	1.32 (0.145)	39.6%	0.36%				0.666
Potassium nitrate	29	1.40 (0.121)	35.8%	−5.82%	−6.20%			

*Note*: Analysis of covariance (ANCOVA) model included baseline and treatment as fixed effect(s), and unequal variances were modeled for each treatment group.

^a^ Two‐sided *p* value comparing treatments using ANCOVA.

A random coefficients model of treatment evaluations on thermal hypersensitivity scores was carried out to assess the benefits measured during the treatment period, which was through Week 8 (see Table 2B). All three treatment groups showed statistically significant improvements versus the negative control (*p* < 0.001). SnF_2_ was significantly more effective than KNO_3_ in this analysis (*p* = 0.015).

**TABLE 2B:** Random coefficients model summary thermal challenge (Schiff Air Index)—evaluable participants.

		*p* value^a^		
Treatment	Adjusted mean (SE)	Stannous fluoride	Oxalate	Potassium nitrate
Negative control	2.30 (0.055)	<0.001	<0.001	<0.001
Stannous fluoride	1.59 (0.065)		0.203	0.015
Oxalate	1.71 (0.062)			0.240
Potassium nitrate	1.81 (0.060)			

*Note*: Random coefficients model included treatment, baseline score, week, and treatment*week.

^a^ Two‐sided *p* value comparing treatments using random coefficients model.

### 3.2 Tactile challenge (Yeaple Probe) sensitivity results

At Day 3 and Weeks 2, 4, 8, and 11, all four groups demonstrated statistically significant reductions (*p* ≤ 0.030) from baseline in tactile scores. Between‐treatment results are shown in Table 3A. At Day 3, the tubule‐occluding treatments—SnF_2_ and oxalate—produced statistically significant improvements in tactile sensitivity compared to the negative control (*p* = 0.001 and *p* = 0.017, respectively), whereas the KNO_3_ treatment produced a nonsignificant 10.7% improvement (*p* = 0.197). The SnF_2_ dentifrice also had a statistically significantly greater tactile score improvement versus the KNO_3_ dentifrice at Day 3 (*p* = 0.020). For Weeks 2 and 4, significant improvements (*p* ≤ 0.019) in tactile sensitivity thresholds versus the negative control continued for all active treatments, with tubule‐occluding treatments providing the largest increases (*p* < 0.001 for both). At Week 8, all active treatments provided significant improvements in tactile sensitivity threshold (*p* < 0.001), basically doubling the pressure required to elicit hypersensitivity response from Day 3. At Week 11, results showed statistically significant durability of benefits in tactile sensitivity threshold (*p* < 0.001) for all three treatment groups—measuring between 73% and 96% increases versus the negative control. Again, results favored tubule occlusion technologies SnF_2_ and oxalate (95%–96%). Tactile sensitivity threshold results are graphically portrayed in Figure 2b.

**TABLE 3A:** Tactile challenge (Yeaple Probe) results by visit. Higher scores indicate less sensitivity.

						*p* value^a^		
Treatment	*n*	Adjusted mean (SE)	% improvement from negative control	% improvement from stannous fluoride	% improvement from oxalate	Stannous fluoride	Oxalate	Potassium nitrate
**Day 3 (baseline mean = 10.588)**								
Negative control	29	12.41 (0.741)				0.001	0.017	0.197
Stannous fluoride	30	16.70 (1.039)	34.6%				0.623	0.020
Oxalate	30	15.90 (1.240)	28.2%	−4.78%				0.131
Potassium nitrate	30	13.74 (0.704)	10.7%	−17.7%	−13.6%			
**Week 2 (baseline mean = 10.588)**								
Negative control	30	14.17 (1.022)				<0.001	<0.001	0.019
Stannous fluoride	30	22.70 (1.987)	60.2%				0.663	0.102
Oxalate	29	21.61 (1.533)	52.5%	−4.82%				0.163
Potassium nitrate	30	18.57 (1.528)	31.0%	−18.2%	−14.1%			
**Week 4 (baseline mean = 10.593)**								
Negative control	30	13.93 (0.988)				<0.001	<0.001	<0.001
Stannous fluoride	30	26.76 (2.548)	92.0%				0.571	0.195
Oxalate	28	25.06 (1.556)	79.8%	−6.35%				0.322
Potassium nitrate	30	22.76 (1.710)	63.3%	−14.9%	−9.17%			
**Week 8 (baseline mean = 10.593)**								
Negative control	30	13.45 (0.824)				<0.001	<0.001	<0.001
Stannous fluoride	30	29.30 (2.728)	118%				0.812	0.581
Oxalate	29	28.49 (2.044)	112%	−2.78%				0.713
Potassium nitrate	29	27.45 (1.926)	104%	−6.31%	−3.63%			
**Week 11 (baseline mean = 10.593)**								
Negative control	30	12.71 (0.664)				<0.001	<0.001	<0.001
Stannous fluoride	30	24.75 (2.631)	94.8%				0.975	0.377
Oxalate	29	24.85 (1.863)	95.6%	0.42%				0.260
Potassium nitrate	29	21.92 (1.801)	72.5%	−11.4%	−11.8%			

*Note*: Analysis of covariance (ANCOVA) model included baseline and treatment as fixed effect(s), and unequal variances were modeled for each treatment group.

^a^ Two‐sided *p* value comparing treatments using ANCOVA.

As with thermal sensitivity measures, a random coefficients model of treatment evaluations was applied to tactile hypersensitivity thresholds (see Table 3B). Random coefficients model analysis showed that across time during the treatment period, all three treatments produced significant increases in tactile sensitivity pressure thresholds (*p* < 0.001), with SnF_2_ treatment producing a directional (*p* = 0.071) improved benefit compared with KNO_3_.

**TABLE 3B:** Random coefficients model summary tactile challenge (Yeaple Probe)—evaluable participants.

		*p* value^a^		
Treatment	Adjusted mean (SE)	Stannous fluoride	Oxalate	Potassium nitrate
Negative control	12.85 (0.996)	<0.001	<0.001	<0.001
Stannous fluoride	21.21 (1.071)		0.549	0.071
Oxalate	20.30 (1.064)			0.229
Potassium nitrate	18.53 (1.013)			

*Note*: Random coefficients model included treatment, baseline score, week, and treatment*week.

^a^ Two‐sided *p* value comparing treatments using random coefficients model.

### 3.3 Complete relief

Analyses were performed on a participant‐level basis examining the proportion of participants that had at least one test tooth (Table 4), as well as both test teeth, transitioning from a thermal score of 2 or greater to 0 (pain‐free) at an ensuing timepoint, indicating complete relief.

**TABLE 4:** Kinetics of complete sensitivity relief in patients during treatment phase.

A. Participant level: number and % of subjects in each group with at least one tooth with ≥2 score at baseline transitioning to 0.					
Treatment	Baseline	Day 3	Week 2	Week 4	Week 8
Negative control	0/30 = 0%	0/29 = 0%	0/30 = 0%	1/30 = 3%	0/30 = 0%
Potassium nitrate	0/30 = 0%	0/30 = 0%	1/30 = 3%	2/30 = 7%	8/29 = 28%
Oxalate	0/30 = 0%	1/29 = 3%	5/29 = 17%	7/28 = 25%	10/29 = 35%
Stannous fluoride	0/30 = 0%	0/30 = 0%	8/30 = 27%	11/30 = 37%	16/30 = 53%

B. *p* value tables: participant level (at least one tooth with ≥2 thermal challenge [Schiff] score at baseline transitioning to 0).			
	*p* value between groups		
Treatment	Potassium nitrate	Oxalate	Stannous fluoride
**Week 2**			
Negative control	1.00	0.024	0.005
Potassium nitrate		0.103	0.026
Oxalate			0.383
**Week 4**			
Negative control	1.00	0.023	0.003
Potassium nitrate		0.075	0.01
Oxalate			0.337
**Week 8**			
Negative control	0.002	<0.001	<0.001
Potassium nitrate		0.570	0.044
Oxalate			0.145

At Week 2, 27% of participants using SnF_2_, 17% using oxalate, and 3% using KNO_3_ exhibited at least one tooth with complete pain relief, compared to 0 out of 30 participants using the negative control. The difference between oxalate and SnF_2_ compared to negative control was statistically significant (*p* ≤ 0.024), while the difference was also significant for SnF_2_ compared to KNO_3_ (*p* = 0.026). At Week 4, 37% of participants using SnF_2_, 25% using oxalate, and 7% using KNO_3_ exhibited at least one tooth with complete pain relief, compared to only 1 out of 30 participants using the negative control. The difference between oxalate and SnF_2_ compared to the negative control was statistically significant (*p* ≤ 0.023), while the difference was also significant for SnF_2_ compared to KNO_3_ (*p* = 0.010). At Week 8, 53% of participants using SnF_2_, 35% using oxalate, and 28% using KNO_3_ exhibited at least one tooth with complete pain relief compared to 0 out of 30 participants using the negative control. The difference between all active treatments compared to the negative control was significant (*p* ≤ 0.002), while the difference was also significant for SnF_2_ compared to KNO_3_ (*p* = 0.044). When defining complete relief as both test teeth that scored ≥2 on the thermal (Schiff) scale at baseline transitioning to a pain‐free score of 0 at an ensuing timepoint, only SnF_2_ at 8 weeks was found to be significantly better than the negative control (*p* = 0.024). The respective transition rates of participants having both teeth reach complete relief at 8 weeks were 20% (6/30) for SnF_2_, 10% (3/29) for oxalate, 3% (1/29) for KNO_3_, and 0% (0/30) for the negative control.

### 3.4 Safety

There were nine adverse events involving eight participants. Four were in the oxalate group and five were in the KNO_3_ group. All were mild in severity and resolved. No participants dropped due to an adverse event.

## 4 DISCUSSION

Technologies to alleviate dentin hypersensitivity are important given it is a common and bothersome condition.^1^ Exposed dentin through periodontal attachment loss as well as gingival recession increases susceptibility to hypersensitivity. The pain associated with hypersensitivity can significantly affect quality of life of patients^24^, ^25^ and can, in principle, discourage proper hygiene procedures leading to the development of additional oral problems. This clinical study is novel in that it addresses key patient‐desired benefits—onset of action, protection against a variety of stimuli, magnitude of benefit, and durability comparing tubule occlusion and nerve depolarization suppression technologies for these clinical benefits.

Considering first onset, tubule‐occluding technologies provided the most rapid sensitivity relief. Day‐3 and Week‐2 analyses showed consistent benefits of SnF_2_ and oxalate technologies compared with the nerve desensitization technology KNO_3_. Desensitizing benefits for KNO_3_ increased in magnitude throughout the clinical period, approaching the tubule‐occluding technologies over time, for both thermal and tactile measures. Through Weeks 4 and 8, participants continued to show improvements in symptoms for all three active treatments, with benefits directionally favoring the tubule‐occluding technologies.

The durability of relief was measured by the assessment of clinical benefits retained following cessation of active treatment at Week 8 and use of the negative control for all participants from Weeks 8 to 11. This is a unique feature of this study design. All three active technologies exhibited significant retention of protection for both cold air and tactile sensitivity thresholds. Durability of thermal benefit was similar for the three active treatments, while tactile threshold measures numerically (11%–12%) favored tubule occlusion technologies (oxalate and SnF_2_) versus KNO_3_ dentifrice.

Protection from different hypersensitivity stimuli is also an important benefit. Three major stimuli for patients include thermal (hot/cold stimulus, often from beverages), tactile sensitivity (often seen during hygiene), and sweet/sour (hygroscopic) foods. In this study, two of these were assessed. All three technologies produced alleviation of hypersensitivity for both thermal and tactile provocation throughout the study. Collectively, the SnF_2_ dentifrice performed the best overall for both types of tooth sensitivity stimuli.

The last efficacy component to be examined was complete relief. Participant‐level analyses examined the proportion of participants on each treatment with at least one test tooth scoring ≥2 on the thermal (Schiff) scale at baseline transitioning to a pain‐free score of 0 at an ensuing timepoint, indicating clinical achievement of complete pain relief on a specific test tooth. It is expected that patients desire complete alleviation of symptoms as a clinically meaningful end point. The results show that the different technologies deliver complete pain relief with substantially different kinetic curves. The first indication of complete relief was observed at Week 2, with oxalate (17% of population) and SnF_2_ (27% of population) providing statistically significantly greater complete relief than the negative control. The proportion of participants experiencing complete relief at Week 4 increased with oxalate (25% of population) and SnF_2_ (37% of population) and again at Week 8 with oxalate (35% of population) and SnF_2_ (53% of population). In contrast, KNO_3_ did not provide statistically significant complete relief until Week 8, with 28% of the population experiencing complete relief. SnF_2_ provided statistically significantly greater complete relief compared to KNO_3_ at Weeks 2, 4, and 8, emphasizing that the kinetic benefits of onset of action are different between these actives. All interim timepoint sensitivity data were used for the time to transition to complete sensitivity relief analysis. The cumulative incidence curve and log‐rank test showed that by Week 8 the SnF_2_ group had the highest proportion of subjects with complete relief followed by experimental oxalate, KNO_3,_ and negative control (see Figure S1 in the online *Journal of Periodontology*). The median transition time to complete relief for the SnF_2_ group was 8 weeks. No other groups had 50% of participants reaching complete relief by Week 8.

Overall, all sensitivity‐oriented treatments produced benefits in alleviating dental pain due to thermal and tactile stimuli in this study. Tubule‐occluding technologies, including experimental oxalate as well as commercial SnF_2_ toothpaste, appeared to work faster and treat tooth sensitivity to a greater extent than the commercial technology based on nerve‐depolarizing KNO_3_. The efficacy of SnF_2_ in this regard is significant since this technology provides additional benefits with respect to both gingival health as well as dental caries. For durability of effect 3 weeks post treatment (Week 11), oxalate, SnF_2_, and KNO_3_ all provided a statistically significant benefit versus the negative control, maintaining 68%–83% for thermal and 66%–81% for tactile of the maximum benefit achieved at 8 weeks. The three treatment groups were not statistically significantly different from one another at Week 11 (*p* ≥ 0.260).

The results of this trial corroborate clinical trials demonstrating efficacy for SnF_2_ formulations in treating dentin hypersensitivity.^26^, ^27^ Multiple randomized controlled trials have shown statistically significantly greater improvements in thermal and tactile sensitivity for SnF_2_ dentifrice compared to sodium fluoride controls, starting as early as first brushing^28^ and extending to 8 weeks of use.^29^, ^30^ Direct comparisons of SnF_2_ formulations to other technologies examining benefit kinetics are not commonplace, therefore these results provide novel findings regarding relative efficacy of common over‐the‐counter dentin hypersensitivity treatments.

Key strengths of this study include use of well‐established clinical methods, assessment of multiple efficacy parameters (e.g., onset, magnitude, completeness, and durability), and consistency of results across different end points. The study population diversity, which is reflective of US population trends, and the use of two common sensitivity stimuli speak to the generalizability of the data. Moreover, 72% of study participants were women, which is consistent with dentin hypersensitivity prevalence data reported elsewhere in the literature.^31^ A limitation of the research is that data extrapolation should be constrained to the timepoints examined and to the products tested since formulation chemistry can impact ingredient stability and/or bioavailability.^32^ Future research could include additional visits to delineate specific efficacy parameters further.

## 5 CONCLUSION

This randomized clinical trial demonstrated significant benefits for SnF_2_, KNO_3_, and oxalate dentifrice treatments versus a negative control in thermal and tactile dentin hypersensitivity reduction over 8 weeks and during a 3‐week relapse phase. The SnF_2_ dentifrice showed the greatest benefits for onset of relief, magnitude of benefit, and complete relief of dentin hypersensitivity.

## AUTHOR CONTRIBUTIONS

**Aaron R. Biesbrock**: Conceptualization; methodology; data interpretation; visualization; writing—original draft. **Tao He**: Data interpretation; visualization; writing—original draft. **Yuanshu Zou**: Conceptualization; methodology; formal analysis; visualization; data interpretation; writing—original draft. **Julie M. Grender**: Conceptualization; formal analysis; visualization; data interpretation; writing—original draft. **Pejmon Amini**: Execution; writing—review and editing. **Paul A. Sagel**: Conceptualization; data interpretation; writing—review and editing. **Andrew Groth**: Conceptualization; execution; writing—review and editing. **Malgorzata Klukowska**: Conceptualization; methodology; data interpretation; writing—review and editing.

## CONFLICT OF INTEREST STATEMENT

Dr. Biesbrock, Dr. He, Dr. Zou, Dr. Grender, Mr. Sagel, Mr. Groth, and Dr. Klukowska are employees of The Procter & Gamble Company. Dr. Amini has no conflicts of interest to disclose.

## Supporting information



Supporting Information

## Data Availability

The datasets generated and analyzed during the current study are not publicly available as they are considered proprietary but may be available from the corresponding author upon reasonable request.

## Footnotes

* Experimental dentifrice; Procter & Gamble, Cincinnati, Ohio, USA

† Sensodyne Extra Whitening; Haleon, Weybridge, England

‡ Crest Pro‐Health Sensitive and Enamel Shield; Procter & Gamble, Cincinnati, Ohio, USA

§ Colgate Cavity Protection; Colgate‐Palmolive, New York, New York, USA

‖ Oral‐B Indicator; Procter & Gamble, Cincinnati, Ohio, USA
