# Supplementary material for: Randomized clinical trial evaluating kinetic benefits of desensitizing agents: Magnitude, onset, and stability of relief
Source: J Periodontol. 2025 May 30;96(12):1339–51. doi: 10.1002/JPER.24-0688 (PMC12819361; doi:10.1002/JPER.24-0688)
Supplement: Supplementary file 1 — Supporting Information [file JPER-96-1339-s001.pdf]

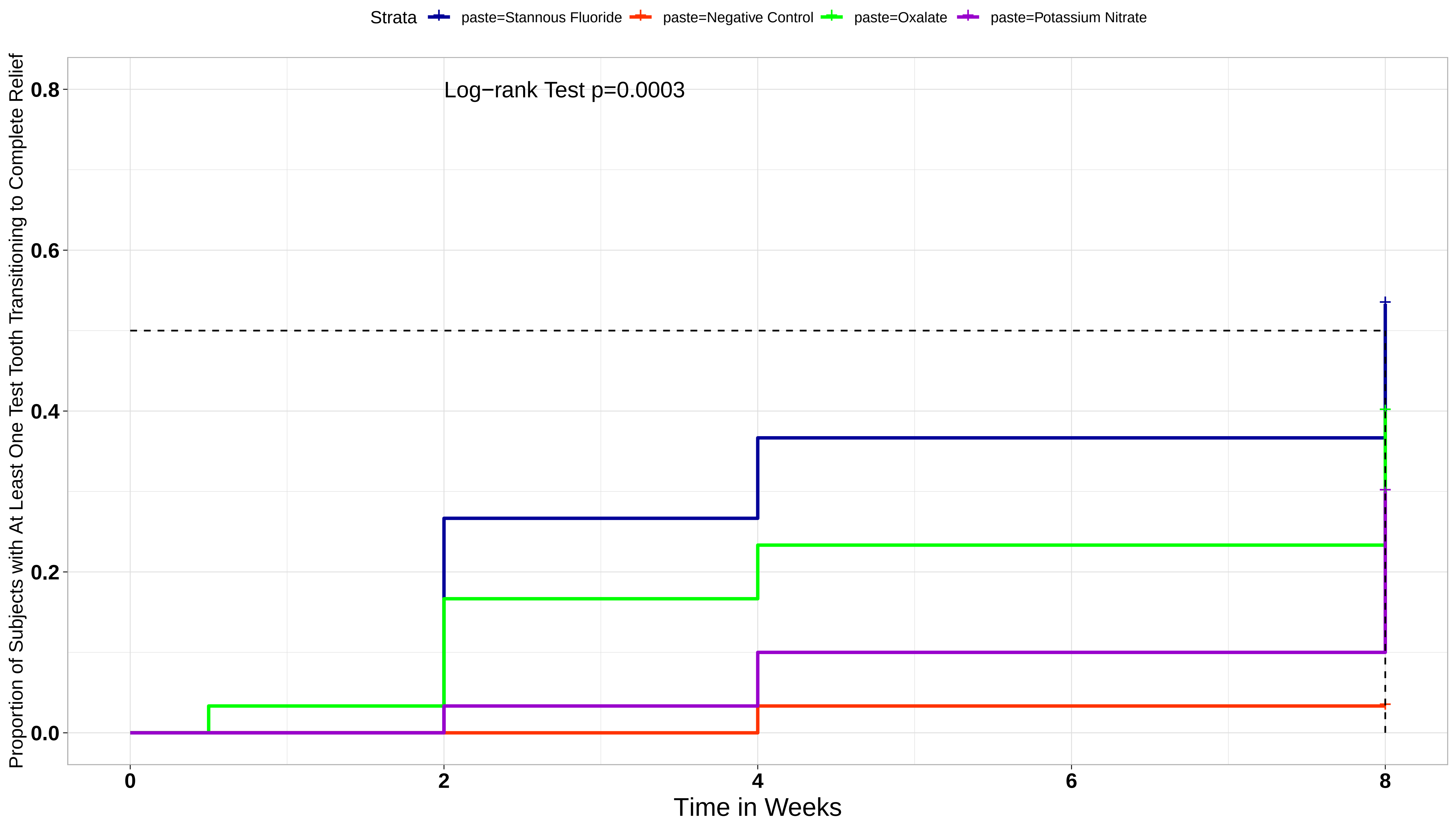

Number for potential moving to sensitivity complete relief (Number of events)

Strata	0	2	4	6	8
paste=Stannous Fluoride	30 (0)	30 (8)	22 (11)	19 (11)	19 (16)
paste=NegativeControl	30 (0)	30 (0)	30 (1)	29 (1)	29 (1)
paste=Oxalate	30 (0)	29 (5)	25 (7)	23 (7)	23 (12)
paste=Potassium Nitrate	30 (0)	30 (1)	29 (3)	27 (3)	27 (9)

Time in Weeks
